# ﻿*Cryptanthaacrimuricata* (Boraginaceae), a distinctive new taxon of series *Muricatae*

**DOI:** 10.3897/phytokeys.250.138635

**Published:** 2024-12-30

**Authors:** Michael G. Simpson, Lee M. Simpson, James M. André

**Affiliations:** 1 Department of Biology, San Diego State University, San Diego, California 92182, USA San Diego State University San Diego United States of America; 2 Granite Mountains Desert Research Center, University of California, Riverside, PO Box 101, Kelso, California 92309, USA University of California Kelso United States of America

**Keywords:** Boraginaceae, *Cryptantha*, *Cryptanthamuricata*, series *Maritimae*, series *Muricatae*, taxonomy

## Abstract

In the process of studying the species *Cryptanthamuricata* and its varieties, we discovered a unique taxon of the genus that resembles *C.muricata* but differs in having a mostly densely white-strigose stem vestiture (sometimes with spreading trichomes) and tuberculate to muricate nutlets with often whitish tubercles that are, in comparison with typical *C.muricata*, larger, with a wider base and more pointed apex, and more densely spaced. We believe this form to be different enough to describe as a new species, *Cryptanthaacrimuricata*. This new species occurs in southwestern North America: in California and Arizona of the United States and in northern Baja California, Mexico. It occurs in mid- to relatively high elevation mountain regions of mostly desert transition/escarpment in the Transverse and Peninsular Ranges, in the Sonoran and Mohave Deserts, and with some populations scattered in the southern Sierra Nevada. We believe this new species to be closely related to *C.clokeyi*, *C.martirensis*, *C.muricata*, and possibly *C.hooveri*, of *Cryptantha* series *Muricatae*. Detailed molecular phylogenetic are needed to better establish their interrelationships.

## ﻿Introduction

The genus *Cryptantha* (Boraginaceae, subtribe Amsinckiinae, after Chacón et al. 2016), as currently delimited, consists of approximately 110 species and 125 minimum-rank taxa, with 64 species native to North America, 47 species native to South America, and one of these found on both continents (see Hasenstab-Lehman and Simpson 2012; Guilliams et al. 2017; Simpson et al. 2017a; Amsinckiinae Working Group 2024). The numerous species in the genus are delimited from one another by duration (perennial versus annual), stem habit/branching pattern and vestiture, inflorescence cymule number, leaf position/morphology, floral bract presence, calyx shape/size/vestiture, corolla shape/size/color, and fruit (nutlet) morphology. The last feature is often most important in taxonomic circumscription. Nutlets of different *Cryptantha* species may vary markedly in several features, including: number per fruit (1–4), heteromorphism [with some species having one nutlet different from the other(s)], size, shape, and sculpturing types; the last feature includes smooth, papillate, muricate/tuberculate, spinulose, and/or winged (see Simpson and Hasenstab 2009).

Recent molecular phylogenetic analyses (Hasenstab-Lehman and Simpson 2012; Simpson et al. 2017b; Mabry and Simpson 2018) have clarified generic circumscriptions within subtribe Amsinckiinae, to which *Cryptantha* belongs (see Chacón et al. 2016). These studies provided evidence for the segregation of *Eremocarya*, *Greeneocharis*, *Johnstonella*, and *Oreocarya* from the traditional concept of *Cryptantha* s.l., which had been circumscribed in having a ventral groove attachment scar. Several taxonomic studies (Mabry et al. 2016; Simpson et al. 2013, 2014, 2016, 2019; Simpson and Kelley 2017; Simpson and Rebman 2013, 2021a, b; Rebman and Simpson 2022; and Simpson and York 2024) have contributed to an understanding and recognition of new species and infraspecies within *Cryptantha* and its close relatives.

In the process of studying the species *Cryptanthamuricata* (Hook. & Arn.) A.Nelson & J.F.Macbr. and its varieties (work in progress), we discovered a unique form that resembles aspects of the three varieties of *C.muricata* in calyx shape and in nutlet shape and presence of a median ridge. However, the new species differs from all *C.muricata* varieties in stem vestiture and in nutlet sculpturing. We believe that these differences warrant its description as a species new to science, based on a taxonomic (morphologic) concept (Cronquist 1978, 1988).

## ﻿Methods

Herbarium specimens of what originally had been identified as *Cryptanthamuricata* from **ARIZ**, **ASU**, **BCMEX**, **BSCA**, **CAS/DS**, **GMDRC**, **HSC**, **JOTR**, **LOB**, **PUA**, **RSA/POM**, **SBBG**, **SD**, **SDSU**, **UC/JEPS**, and **UCR** (herbarium acronyms after Thiers 2024) were examined, with focus on nutlet morphology. This new species was identified as distinct from the three recognized varieties of *Cryptanthamuricata* (Table 1), after comparative study of those specimens. In order to verify that this entity had not been previously named, we studied online images of type specimens of all heterotypic synonyms of *Cryptanthamuricata* varieties: *C.horridula* Greene, *C.densiflora* A.Nelson & P.B.Kenn., and *C.vitrea* Eastw. (Table 1; see Amsinckiinae Working Group 2024).

**Table 1.** *Cryptantha* series *Muricatae*. Diagnosis and classification, with 5 species and 7 minimum-rank taxa. Heterotypic synonyms, only occurring for the varieties of *C.muricata*, are also listed. *=Included in the molecular phylogenetic studies of Simpson et al. 2017 and Mabry and Simpson 2018. †=Placement tentative.

Series *Muricatae*: Nutlets 4 per fruit, homomorphic, ovate to triangular, papillate and turberculate to muricate, style extending beyond nutlets at maturity
*Cryptanthaacrimuricata* J.M.André, L.M.Simpson, & M.G.Simpson
*Cryptanthaclokeyi I.M.Johnst., J. Arnold Arbor. 20: 387 (1939)
†*Cryptanthahooveri* I.M.Johnst., J. Arnold Arbor. 18: 23 (1937)
*Cryptanthamartirensis M.G.Simpson & Rebman, Madroño 60: 35 (2013)
*Cryptanthamuricata(Hook. & Arn.)A.Nelson & J.F.Macbr.var.muricata, Botanical Gazette 61: 42 (1916)
[*Cryptanthahorridula* Greene, Pittonia 5: 55 (1902)]
Cryptanthamuricatavar.denticulata (Greene) I.M.Johnst., Contr. Gray Herb. 74: 71 (1925)
[*Cryptanthadensiflora* A.Nelson & P.B.Kenn., Proc. Biol. Soc. Washington 19: 156 (1906)]
Cryptanthamuricatavar.jonesii (A.Gray) I.M.Johnst., Pl. World 22: 114 (1919)
[*Cryptanthavitrea* Eastw., Proc. Calif. Acad. Sci., ser. 3, 2: 292 (1900)]

All known specimens corresponding to this new species were assembled, and their associated collection data were collated in a spreadsheet. A similar spreadsheet was prepared using selected specimen data for the three varieties of *Cryptanthamuricata*. Point distribution maps, at two scales, were prepared of the localities of these collections, the georeference data either recorded directly from herbarium sheet label data or estimated from recorded locality information of these labels. Maps were prepared using the Berkeley multi-mapper tool (https://ucjeps.berkeley.edu/consortium/load_mapper_multi.html). Stem and leaf morphology, mature (fruiting) calyx, and nutlets of selected specimens were photographed from field observations and from herbarium specimen material. High magnification images were produced using a Macropod Pro 3D camera system (Macroscopic Solutions, East Hartford, CT, USA) or an Infinity 2 camera on an Olympus SZ61 boom-mounted dissecting microscope. All specimens were studied in generating a description of the new taxon. A key was prepared to separate this new species from *Cryptanthamuricata* and close relatives.

## ﻿Taxonomic treatment

### ﻿ Cryptantha acrimuricata

Taxon classificationPlantae

J.M.André, L.M.Simpson, & M.G.Simpson sp. nov.

DCA50A96-3FBB-5746-B343-16561BB29571

urn:lsid:ipni.org:names:77354339-1

#### Type.

United States • California, San Bernardino County, New York Mountains: Mojave Natl. Preseve; Caruthers Canyon, 2.3 road miles north of junction New York Mtns Rd, at base of steep ascent to Giant Ledge Mine (historic), along closed dirt road paralleling perennially moist drainage, California, San Bernardino Co., 35.24875, -115.299217, 1768 m elevation, pinyon-juniper-oak woodland, among granitic outcrops, associates: *Pinusmonophylla*, *Quercusturbinella*, *Garryaflavescens*, *Arctostaphylospungens*, *Bouteouagracilis*, *Yuccabaccata*, *Brickelliacalifornica*, *Opuntiaphaeacantha*, *Dudleyasaxosaaloides*, *J. M. André 40457*, 10 May 2019 (**holotype: RSA**0633491; **isotypes: ASU**, **GMDRC**, **SBBG**, **SD**, **SDSU**, **UC**, **UCR**).

#### Diagnosis.

*Cryptanthaacrimuricata* resembles *C.muricata* and varieties in having ovoid fruiting calyces with nutlets that are typically four per fruit, homomorphic, ovate in shape, with margins thickened and bearing tuberculate to muricate processes, with a median dorsal ridge, and with surfaces densely tuberculate to muricate. *Cryptanthaacrimuricata* differs from *C.muricata* and varieties in having dense whitish-strigose stems, with spreading trichomes absent to sparsely present, and in having nutlets with relatively large and densely spaced, conic, often whitish, muricate processes with relatively wide bases and sharp apices, surfaces between the processes often shiny.

#### Description.

**Figure 1.**
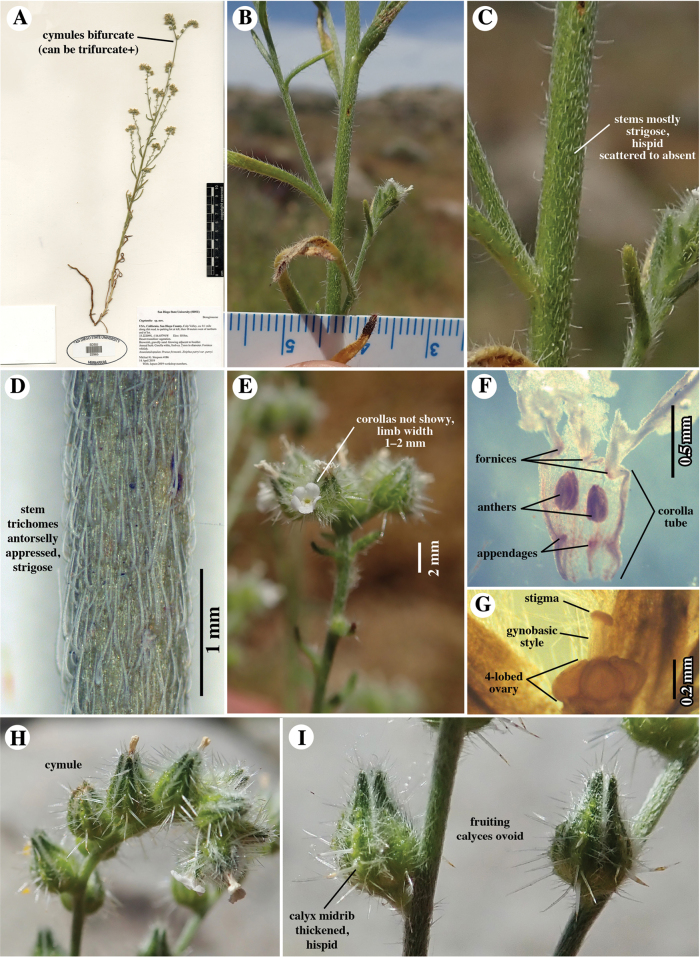
*Cryptanthaacrimuricata*. Images of collection: *M.G. Simpson 4186*, 14 April 2019, **SDSU**22965 **A** herbarium specimen. Note dominant primary stem terminating in bifurcate cymules **B–E, H, I** field photographs **B** primary stem, showing cauline leaves and lateral branches **C** close-up of primary stem, the vestiture primarily strigose plus scattered spreading-hirsute **D** extreme close-up of primary stem, showing predominantly whitish strigose vestiture (trichomes antrorsely oriented) **E** close-up of cymule tip, showing relatively small corolla, this one slightly < 2 mm wide **F, G** flower removed and dehydrated **F** corolla, showing fornices, anthers adnate to corolla tube, and basal appendages **G** gynoecium, showing 4-lobed ovary, gynobasic style, and discoid stigma **H** cymule of primary stem **I** close-up of mature fruiting calyces. Note ovoid shape and spreading-hispid, pustulate trichomes of thickened sepal midribs.

**Figure 2.**
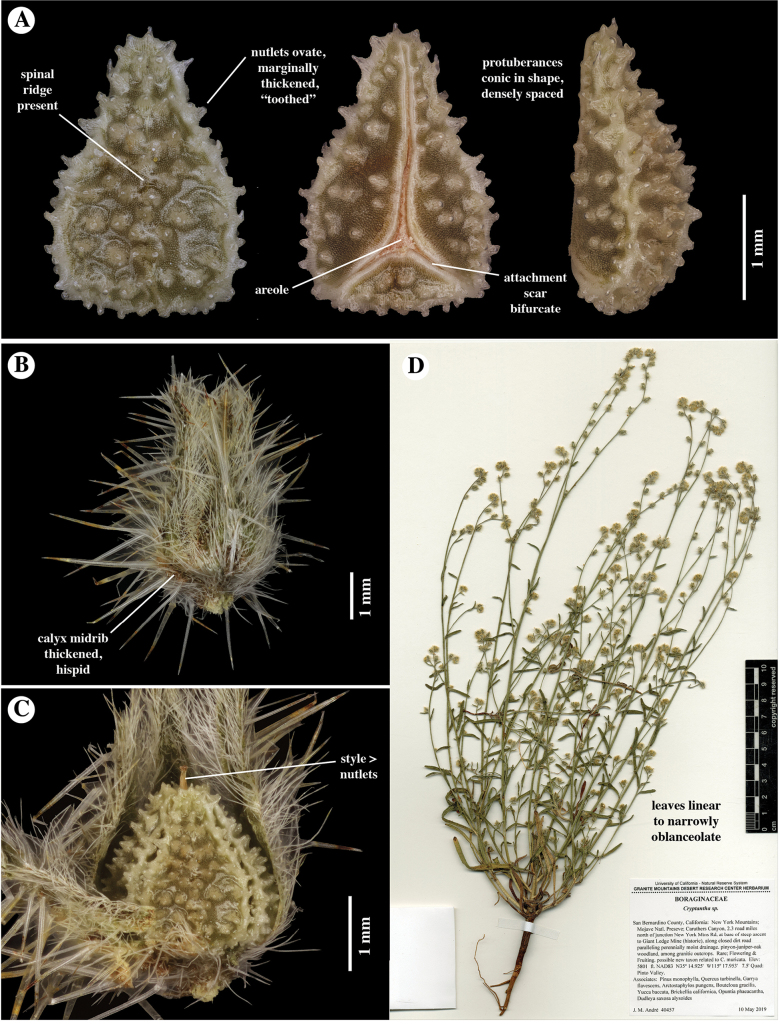
*Cryptanthaacrimuricata*. Images from holotype specimen: *J.M. André 40457*, 10 May 2019, **RSA**0633491 **A** nutlet, in (left to right) dorsal, ventral, and lateral views. Note ovate shape (dorsal and ventral outlines); truncate base; thickened, “toothed” or “beaded” margins; whitish, conic surface tubercles; and narrow ventral groove, which is widely bifurcate at the base, delimiting small areole **B** fruiting calyx, showing appressed marginal trichomes and spreading hispid along midrib **C** fruit opened, showing prominent style, protruding well beyond nutlets **D** herbarium specimen.

(Figs 1, 2). ***Plants*** annual herbs, 10–35 cm tall, mostly gray-green. ***Root*** a taproot, brown to red-purple, sometimes staining paper. ***Stems*** generally erect to ascending, some plants with a dominant, erect primary axis bearing several short to elongate, ascending, secondary branches, others with numerous, subequal, inclined to ascending branches arising from plant base; stem surface light green, vestiture strigose with dense, white, tapered, antrorsely appressed, basally white-pustulate trichomes, ca. 0.5–0.7 mm long; hispid vestiture scattered to absent, if present with few, white, tapered, spreading (horizontal to inclined), basally white-pustulate trichomes, ca. 1 mm long. ***Leaves*** mostly gray-green, sessile, linear to narrowly oblanceolate, midrib grooved adaxially, thickened abaxially, apex rounded to obtuse, both surfaces and margin appressed to ascending hirsute, trichomes white pustulate, especially in basal leaves; basal-most leaf pair apparently opposite, perhaps remnants of cotyledons, all others alternate-spiral; basal leaves 2–5 cm × ca. 2–3 mm, in a loose rosette withering at anthesis, cauline leaves 1–2 cm × ca. 1 mm, extending to base of cymule inflorescence units, slightly reduced upwards. ***Inflorescence*** cymules mostly in clusters of 2–3 (bifurcate or trifurcate), occasionally solitary, rarely up to 5, a terminal flower/fruit typically at junction of cymule bases; bracts occasionally at base of cymules, these linear to narrowly oblong; cymules elongated in fruit, 2–12 flowers per unit, lower fruits not touching at maturity. ***Flowers*** mostly ebracteate, pedicellate, pedicels in fruit more elongate, ascending, thickened apically, 0.5–1 mm long, surface densely hirsute, trichomes horizontal to ascending. ***Calyx*** symmetric, ca. 3 mm long at anthesis, sepals distinct; fruiting calyx ovoid, base truncate, apex acuminate, 3.5–5 mm long, sepals lance-ovate to lanceolate, apices erect; abaxial surface densely hirsute ascending along margins, marginal surfaces hirsute appressed, midrib strongly thickened, hispid, the trichomes stout, straight, tapered, pustulate, inclined, horizontal, or reflexed; adaxial surface glabrous basally, appressed short hirsute on surface of extreme apex. ***Corolla*** rotate, inconspicuous, white, fornices white, tube as long as calyx, limb 1–2 mm wide, not showy, appendages present ca. 1/3 from base of corolla tube in vertical line with anthers. ***Androecium*** of five stamens, attached at slightly different levels ca. 2/3 from base of corolla tube between and below fornices; anthers ca. 0.4 mm long, ellipsoid, dithecal, introrsely dehiscent, dorsifixed; filaments filiform, ca. 0.1 mm long. ***Gynoecium*** four-lobed, lobes ca. 0.3 mm long, globose, style gynobasic, ca. 0.7 mm long, protruding ca. 0.4 mm beyond ovary; stigma minute, discoid. ***Gynobase*** narrowly pyramidal, extending nearly to nutlet apices; style extending 0.1–0.3 mm beyond nutlet apices. ***Nutlets*** 4, erect, homomorphic, ca. 2–2.4 mm × 1.7 mm (at widest width), whitish to tan, sometimes dark brown-mottled, surface typically shiny, appearing varnished, ovate, abaxially flattened to low convex, adaxially shallowly 2-planed-convex, base truncate, margins sharp-angled with a thickened, usually whitish rim appearing “beaded”/”toothed” from marginal tuberculate to muricate protuberances, nutlet apex acute-rounded (blunt), surface irregularly transversely ridged, papillate and densely white tuberculate to muricate, these processes generally widely conic, densely spaced on surface, especially abaxially; spinal ridge present, sometimes obscure; attachment scar edges abutted at upper two-thirds, strongly bifurcate in lower one-third, delimiting a narrowly triangular, basal areole.

#### Distribution and habitat.

**Figure 3.**
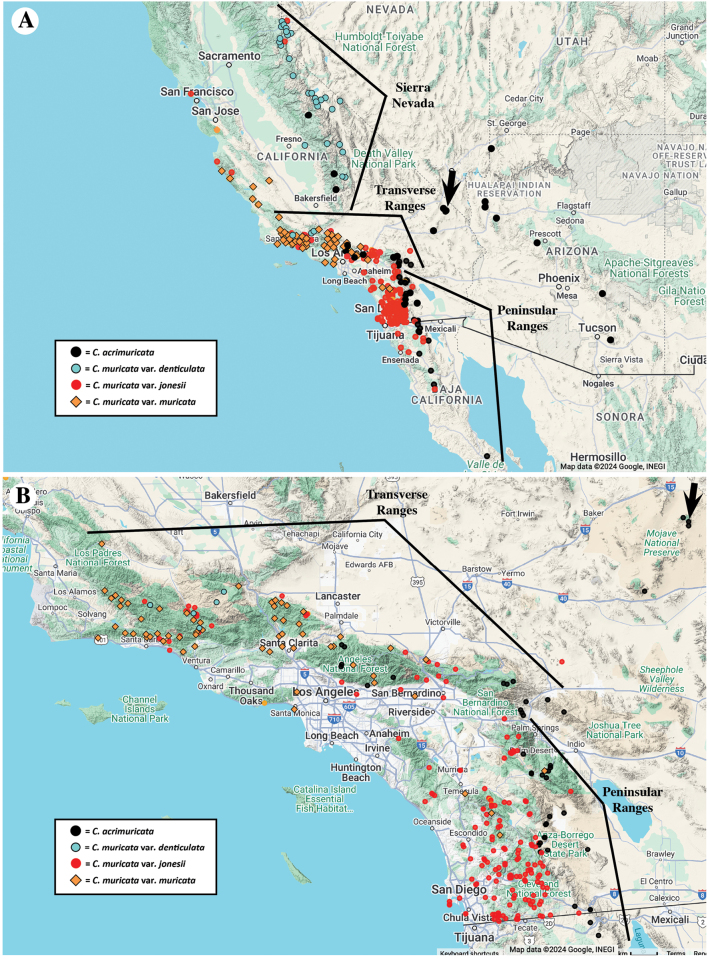
Distribution maps of *Cryptanthaacrimuricata* from specimens cited here, plus of the three varieties of *C.muricata* from preliminary data, in two scales **A** showing overall distribution and **B** in a larger scale map highlighting the Transverse Ranges and northern Peninsular Ranges. See text for distribution summaries of the taxa. Arrow indicates location of holotype/isotypes specimens of *Cryptanthaacrimuricata*. Map data @2024 Google, INEGI.

*Cryptanthaacrimuricata* occurs in the eastern part of the Peninsular Ranges, in the eastern and central Transverse Ranges, in higher elevations of the Mohave and Sonoran Deserts, and in scattered regions of the Sierra Nevada, of California and Arizona in the United States and Baja California in Mexico (Fig. 3). Its habitat ranges from sandy to coarsely gravelly substrates, usually granitic, sometimes limestone, sometimes adjacent to boulders, of chaparral, desert scrub/wash, pinyon-juniper woodland, or pine/oak woodland vegetation. The documented elevation range is 180–2621 m (average 1270 m).

#### Phenology.

Based on data from available specimens, the species typically flowers April–July, rarely in the fall following warm-seasonal rains (J. M. André, personal observation).

#### Rarity and conservation status.

*Cryptanthaacrimuricata* has been somewhat sparsely collected compared to the close relative *Cryptanthamuricata*. Where encountered, populations of *C.acrimuricata* tend to be relatively isolated and restricted in areal extent, with few total numbers of individual plants. Though we are not aware of any imminent threats to existing populations, based upon its fairly limited distribution and small populations, this taxon may warrant conservation status.

#### Etymology.

The specific epithet *acrimuricata* means “sharply muricate,” from the Latin *acri*-, sharp, and *muricata* (derived from Latin *murex*, conch), hard-pointed. The name refers to the stout, widely-based, and sharply pointed conic tubercles, shaped like miniature mountain peaks, as contrasted with the more cylindrical or rounded tubercles of the varieties of *Cryptanthamuricata*.

#### Suggested common name.

We suggest Sharp-Pointed Prickly Cryptantha as a common name.

##### ﻿Paratypes

Arranged by country, then alphabetically by collector; *=estimated from label data.

Mexico • Baja California. San Rafael, Sierra San Pedro Mártir; west side of Cerro Blanco; 3k SSW of San Rafael, Baja California, 31.09333, -115.65167, 1550 m elevation, west facing slope, granite rock and gravel, associates: *Adenostomafasciculatum*, *Arctostaphylos*, *Chorizanthefimbriata*, *Eriogonumfasciculatum*, *Lotusrigidus*, *Lupinusconcinnus*, *Mimuluspilosus*, *Rhusovata*, *Salviaapiana*, *Swertiaalbomarginata*, and *Trichostemmaparishii*, *M. A. Baker 12915 with Robert Johnson*, 12 May 1998 (**ASU**0014635!, **BCMEX**11466!); • Cerro San Luis, Baja California, 29.3167, -114.1167, 1300 m elevation, open gravelly place, north side, gravelly, *R. Moran 10296 with Jim Henrickson*, 2 March 1963 (**RSA**165004!, **SD**54579, **UC**1235600); • Sierra Juarez, 5 km W of La Rumorosa, Baja California, 32.55, -116.1, 1325 m elevation, colony under piñon, granitic area, associates: Piñon, *R. Moran 24108*, 15 May 1977 (**CAS**612817!, **SD**97068!); • Sierra Juarez, 1 km NW of Tres Pozos, Baja California, 32.37917, -116.075, 1350 m elevation, under shrubs along arroyo, *R. Moran 27383*, 26 May 1979 (**RSA**292842!, **SD**103648!); • Sierra Juarez, 1.5 km NW of El Mezquite, Baja California, 32.35833, -116.0667, 1400 m elevation, semishade of piñones, piñon-juniper-pine wood, associates: piñon-juniper-pine wood, *R. Moran 27437*, 27 May 1979 (**POM**369185!, **SD**103689); • Sierra Juarez, arroyo Agua Grande, 10 km south of La Rumorosa, Baja California, 32.4625, -116.0333, 1225 m elevation, in semishade, among rocks, *R. Moran 30751*, 16 May 1982 (**SD**110999!); • San Salvador: Bridge over branch of Rio San Carlos near Rancho San Salvador, Baja California, 31.8333, -116.0667, 1000 m elevation, *R. F. Thorne 60086 with Dave Charlton*, 19 April 1985 (**RSA**346809!); • Canon de Guadalupe, Baja California, 32.15, -115.8, 450 m elevation, dry slopes along canyon above resort area, sandy areas and rocky, *R. F. Thorne 61750 with Steve Boyd*, *etc.*, 23 March 1986 (**RSA**349247!); 1.5 mi NW Village of Valle Trinidad, Baja California, 31.423618*, -115.739987*, 989 m elevation*, granitic hillside, *I. L. Wiggins 16061A*, 3 April 1960 (**DS**506948!);

United States • New York Mtns, [above Brant Siding] in canyon above old mine site, California, San Bernardino Co., 35.27667, -115.34556, 1364 m elevation, granite, talus, gravel, *J. M. André 4153 with G.L. Clifton*, 7 May 2003 (**GMDRC**1194!, **UCR**-164170!); • New York Mountains: growing in bottom of main drainage, just above main parking area/camp. Caruthers Canyon, Mojave National Preserve, California, San Bernardino Co., 35.224817, -115.303467, 1675 m elevation, at base of boulders in creek bottom, granite, alluvium, gravel, associates: *Salixexigua*, *Baccharissergiloides*, *Carexalma*, *Ericameriacuneata*, *Pinusmonophylla*, *J. M. André 9554 with T. La Doux*, *G.L. Clifton*, 5 May 2008 (**GMDRC**2793!, **SDSU**18622!); • Joshua Tree National Park, Little San Bernardino Mountains: Long Canyon, up side canyon approx. 400 m. west of main canyon drainage, California, Riverside Co., 34.063517, -116.443033, 1172 m elevation, south-facing slope, decomposed granite, alluvium, associates: *Juniperuscalifornica*, *Ephedranevadensis*, *Coleogyneramosissima*, *Mentzeliainvolucrata*, *Calycoserisparryi*, *Eriophyllumconfertiflorum*, *J. M. André 21088 with T. La Doux*, *R.B. Kelley*, 4 May 2011 (**GMDRC**5506!, **GMDRC**5507!, **UCR**0005646!); • Hualapai Mountains: along dry creekbed at Moss Wash trailhead, just below Wild Cow Campground, 4.0 mi. south of Hualapai Mtn Ranger Sta., Arizona, Mohave Co., 35.064183, -113.8672, 1836 m elevation, yellow pine - oak woodland, granite, alluvium, gravel, associates: *Pinusponderosa*, *Quercusturbinella*, *Q.gambelii*, *Q.chysolepis*, *Pteleatrilobata*, *Symphoricarposrotundifolius*, *Prunusvirginiana*, *Eriogonumdavidsonii*, *J. M. André 30870*, 29 May 2014 (**ASU**0307471!, **GMDRC**6806!); • Cerbat Mountains: west side of range c. 12 mi north of Kingman, 0.3 mi along faint dirt road north of Mineral Park Road, 2.7 mi east of Hwy 93, Arizona, Mohave Co., 35.35557, -114.17613, 1166 m elevation, upper alluvial fan, mixed, alluvium, gravel, associates: *Juniperuscalifornica*, *Coleogyneramosissima*, *Larreatridentata*, *Salviadorrii*, *Acamptopappussphaerocephala*, *Krameriaerecta*, *Camissoniopsispallida*, *J. M. André 32653*, 24 April 2015 (**GMDRC**7523!, **UCR**-275600!); • Granite Mountains; UC GM Desert Research Center; upper Granite Cove in wash 0.1 mile south of Granite Cove Spring, 0.2 mile north of Staples cabin, California, San Bernardino Co., 34.786, -115.65863, 1338 m elevation, under granite boulder, sand, associates: *Acaciagreggii*, *Ambrosiaeriocentra*, *Prunusfasciculata*, *Eriophyllumwallacei*, *Plagiobothrysarizonicus*, *Descurainiapinnata*, *J. M. André 33209*, 4 April 2015 (**GMDRC**7681!, **RSA**0087609!, **UCR**0004260!); • Peninsular Ranges: along north side of Hwy 74 approx 1 mile east of Pinyon Flats, south facing slopes of Sugarloaf Mtn, California, Riverside Co., 33.583617, -116.437467, 1210 m elevation, granitic gravelly soils among boulders, granite, alluvium, gravel, associates: *Juniperuscalifornica*, *Adenostomasparsifolium*, *Arctostaphylosglauca*, *Cercocarpusbetuloides*, *Mentzeliaveatchiana*, *Phaceliafremontii*, *Saltugilialatimeri*, *J. M. André 41103*, 5 April 2019 (**GMDRC**11665!); • Peninsular Ranges: southeastern end of San Jacinto Mountains, along Hwy 74, 12.6 miles SW of Palm Desert (Hwy 111), 1.2 miles NNE of Sugarloaf Mtn, California, Riverside Co., 33.603767, -116.4199, 1081 m elevation, granitic boulders, granite, alluvium, gravel, associates: *Pinusmonophylla*, *Rhusovata*, *Ericamerialinearifolia*, *Enceliaactoni*, *Phaceliadistans*, *Chaenactisfremontii*, *Acmisponargophyllus*, *Cryptanthalepida*, *J. M. André 42565*, 14 April 2020 (**GMDRC**12668!); • Peninsular Ranges; Anza Bench region: Hills north of Warner Springs, Indian Flats Campground of Cleveland National Forest along Road 9S05, California, San Diego Co., 33.349, -116.661, 1097 m elevation, slopes and shallow draws bordered by *Quercusagrifolia*, thin soils over granitic bedrock with extensive boulder outcrops and on sandy benches, associates: *Adenostomasparsifolium*, *Ceanothusgreggii*, *C.perplexans*, *Adenostomafasciculatum*, *Gutierreziasarothrae*, *Muhlenbergiarigens*, *Lonicerasubspicata*, *Rosacalifornica* and *Rhustrilobata*, *S. Boyd 11034 with LeRoy Gross*, 4 May 2004 (**RSA**726843!, **UCR**0004357); • Kernville, California, Kern Co., 35.755333*, -118.425998*, 812 m elevation*, *T. S. Brandegee s.n.*, 13 May 1892 (**UC**79312!); Canon wash, 1 mi w of Morengo, California, San Bernardino Co., 34.047071*, -116.621009*, 898 m elevation*, *J. R. Bruff 99a*, 2 April 1928 (**RSA**699336!); • Local landmark: Cerbat Mountains, Chloride Quad, Arizona, Mohave Co., 35.464472, -114.179333, 1584 m elevation, Rocky Hillside; Slope Aspect: North, Slope Position: Lower Third; Vertical Slope Shape: Smooth; Horizontal Slope Shape: Smooth, *G. L. Clifton 14473*, 28 April 1986 (**PUA**32315(Card#64080)!); • Whitewater Canyon Rd. (exit from Interstate-10 ca. 2.5 mi. W of jct I-10 with State 62); near start of Angeles [Pacific] Crest Trail, California, Riverside Co., 33.99178*, -116.663304*, 610 m elevation, riparian woodland (through creosote bush scrub) along rocky sandy stream channel joining the main Whitewater wash, rocky, sandy, associates: *Enceliafarinosa*, *Baccharis*, *Quercus*, *C. Davidson 5578 with B. Gustafson*, 7 April 1977 (**RSA**499703!); • Near Dos Palmas Spring, San Jacinto Mts, California, Riverside Co., 33.61841*, -116.424376*, 914 m elevation, *H. & M. Dearing 4851*, 29 March 1942 (**SBBG**6266!); • Rincon Mountains, Chimenea Canyon above Madrona Ranger Station, Arizona, Pima Co., 32.183772*, -110.593461*, 1219 m elevation, riparian forest in desert scrub, associates: *Quercus*, *Juglans*, *Fraxinus*, *Baccharis*, *Ambrosia*, *M. Fishbein 2013*, 15 April 1994 (**ARIZ**315946!); • South fork of San Joaquin river, California, Madera Co., 37.437614*, -119.239026*, 2621 m elevation, *H. M. Hall 650B with H. P. Chandler*, July 1900 (**UC**79530!); 0.5 west of Peg Leg monument along Henderson Canyon Road, Anza Borrego Desert State Park, California, San Diego Co., 33.3004, -116.314, 180 m elevation*, sandy wash, sandy wash, overlain with dry silty ash from 2003 fire above Coyote Canyon, associates: *Larreatridentata*, *Atriplexlentiformis*, *Phaceliabrachyloba*, *Melilotusindica*, Palafoxiaaridavar.arida, *Geraeacanescens*, Abroniavillosavar.villosa, *Antirrhynumcoulterianum*, *Calycoserisparryi*, *Chaenactisartemisiifolia*, *L. Hendrickson 53 with L. Louise Jee*, 9 April 2004 (**SD**161807!); • San Felipe Valley. Cigarettes Hills. San Felipe Wildlife Area, California Department of Fish & Game property. 1.8 air miles W of SW intersection of State Hwy. 78 and County Rd S-2 and .7 mile N of hwy 78, on south side of prominent hill (2765), California, San Diego Co., 33.0963, -116.5064, 769 m elevation, decomposing pegmatite dike, coarse gravel and sandy soil, associates: *Agavedeserti*, *Juniperuscalifornica*, *Prunusfremontii*, *Ziziphusparryi*, *Ericameriabrachylepsis*, Eriogonumfasciculatumvarpolifolium, *L. Hendrickson 3928*, 10 May 2009 (**BSCA**1213, **SD**214894!); 5 mi. se. of Nevada-Arizona border along Lime Kiln Canyon BLM rd. 242, Arizona, Mohave Co., 36.680548*, -114.009796*, 1000 m elevation, mixed desert shrub community, gravelly limestone soils, associates: *Quercus*, *Juniperus*, *L. C. Higgins 25212*, 6 April 2004 (**BRYV**0155702, **HSC**202913!, **KHD**00031415); • Lime Kiln Canyon, Virgin Mountains, Arizona, Mohave Co., 36.646315, -114.019191, 1250 m elevation, Pinyon, juniper, oak community, limestone soil., *L. C. Higgins 25402 with G. Green*, 4 May 2004 (BRYV0155701, **RENO**81684!, **UNM**0026372); • Arizona, Skull valley, Arizona, Yavapai Co., 34.504684*, -112.687251*, 1311 m elevation, *M. E. Jones 7028*, 28 April 1903 (**UC**78720!); • Arizona, Skull valley, Arizona, Yavapai Co., 34.504684*, -112.687251*, 1311 m elevation, *M. E. Jones s.n.*, 1 May 1903 (**POM**71271!); • Arizona, Skull valley, Arizona, Yavapai Co., 34.504684*, -112.687251*, 1311 m elevation, *M. E. Jones s.n.*, 1 May 1903 (**POM**71274!); • Pinal Mts. above road to Madera Peak on road to Signal Peak and Pinal Peak, Arizona, Gila Co., 33.2822*, -110.821*, 2384 m elevation*, upper south facing slopes, *D. Keil 4628*, 12 May 1969 (**ASU**69306!); • Little San Bernardino Mountains, ridge along Upper East Deception Canyon, Joshua Tree National Park, California, Riverside Co., 33.97936, -116.30985, 1319 m elevation, associates: *Yuccaschidigera*, *Juniperuscalifornica*, *Eriogonumwrightiinodusum*, *Peucephyllumschotii*, *T. LaDoux 3724 with E. Babich*, *N. Pietrasiak*, 21 April 2008 (**JOTR**00914!); 2 miles east of Banner on San Felipe Wash, California, San Diego Co., 33.083288*, -116.516252*, 773 m elevation, *K. McCully 92*, 17 May 1925 (**POM**97231!); • Traverse Ranges; San Bernardino Mountain region: Whitewater Canyon; Along Whitewater River drainage and adjacent slopes below Whitewater Visitor Center (Whitewater Trout Farm) in the vicinity of Bonnie Bell, California, Riverside Co., 33.95477, -116.64248, 525 m elevation, riparian woodland along water course to desert sage scrub on adjacent flats and slopes, *O. Mistretta 4627 with Duncan Bell*, *Jill Beckman*, *Joy England*, *Katie Kane*, *Jamie Hall*, *Chris McDonald*, *Lucila Reccia*, *Lonnie Rodriguez*, *April Sall*, *Tracy Tennant*, 12 April 2010 (**RSA**772061!); • Traverse Ranges; San Bernardino Mountain region: Whitewater Canyon; Along Whitewater River drainage and adjacent slopes below Whitewater Visitor Center (Whitewater Trout Farm), California, Riverside Co., 33.97538, -116.65119, 600 m elevation, riparian woodland along water course to desert sage scrub on adjacent flats and slopes, *O. Mistretta 4702a with Duncan Bell*, *Jill Beckman*, *Joy England*, *Katie Kane*, *Jamie Hall*, *Chris McDonald*, *Lucila Reccia*, *Lonnie Rodriguez*, *April Sall*, *Tracy Tennant*, 12 April 2010 (**RSA**771786!); • Northwest slope of Santa Rosa Mtns., California, Riverside Co., 33.555223*, -116.487153*, 2134 m elevation, dry banks, northwest slope, *P. A. Munz 15086*, 30 May 1937 (**BRYV**0155683, **RSA**0079225, **UC**662529!); • Pines to Palms Highway #74, 15 miles west of Palm Desert, California, Riverside Co., 33.581968*, -116.459557*, 1219 m elevation, *D. Myrick 833*, 30 April 1964 (**SBBG**28428!); • Colorado Desert, California, San Diego Co., 33.05*, -116.12*, m elevation, *C. R. Orcutt s.n.*, April 1889 (**UC**280510!); Walker Canyon Ecological Preserve: between Boulevard and Jacumba on the north side of Interstate 8, along arroyo bottom near stream, California, San Diego Co., 32.66306, -116.22333, 915 m elevation, Chaparral/desert transition, granitic substrates, associates: *Quercuscorneliusmulleri*, *Adenostomasparsifolium*, *Rhusovata*, *Juniperuscalifornica*, *Cylindropuntiaganderi*, *J. P. Rebman 8530 with Jeannie Gregory*, 17 April 2003 (**SD**159509, **UC**1787644!, **UCR**0004378); • San Jacinto Mountains, along the trail toward Spitler Pk from the Hurkey Crk camp to Bonita Vista Rd, California, Riverside Co., 33.6961*, -116.6392*, 1707 m elevation, along trail; chaparral & some yellow pine forest, *A. C. Sanders 6537*, 1 June 1986 (**DES**00030658, **OBI**131801, **SBBG**87639!, **UCR**0004196, **UNLV**23020); • Along FS road 22S82, east base of the Needles, approx. 3 miles from Pyles Camp., California, Tulare Co., 36.10939*, -118.484532*, 2419 m elevation, granitic sandy soils, *J. R. Shevock 5114*, 18 June 1976 (**LOB**100364!); • SW Imperial Co, near SE boundary of San Diego Co., In-Ko-Pah Mountains, access road to Valley of the Moon, California, Imperial Co., 32.639288*, -116.100194*, 1031 m elevation, transition area from scrub oak-yucca to diverse high desert scrub, steep N-facing bouldery drainage, rocky slopes, steep N-facing bouldery drainage; granitic outcrops and soils, mostly coarse particle size;, associates: C.pterocaryavar.cycloptera, C.p.var.purpusii, *C.clevelandii*, *C.intermedia*, *Microsersisdouglasii*, *Giliacaruifolia*, *Phaceliadistans*, *Lotusscoparius*, *Quercuscornelius-muelleri*, *Yuccaschidigera*, *Nolinaparryi*, *Rhusovata*, *Simmondsiachinensis*, *Acaciagreggii*, *Enceliaactoni*, *Salviaapiana*, *Cylindropuntiaganderi*, *C.wolfii*, *Ericameriabrachylepsis*, and Lupinusexcubitusvar.medius, *D. Silverman 3964 with Jon Rebman*, 23 April 2001 (**JEPS**103093!, **SBBG**116573!); • Wash adjacent to and just west of jeep road, Rodriguez Canyon, ca. 2.0 miles southwest of Granite Mtn. peak., California, San Diego Co., 33.03111, -116.50361, 946 m elevation, wash adjacent to exposed, sparse desert scrub, sandy, gravelly soil, associates: *Prunusfremontii*, *Rhusovata*, *Baccharissergiloides*, *Quercus* sp., *M. G. Simpson 2790 with K. Hasenstab*, *M. Silveira*, *and L. Simpson*, 7 April 2007 (**SDSU**19296!); • Wash adjacent to and just west of jeep road, Rodriguez Canyon, ca. 2.0 miles southwest of Granite Mtn. peak., California, San Diego Co., 33.03111, -116.50361, 946 m elevation, wash adjacent to exposed, sparse desert scrub, sandy, gravelly soil, associates: *Prunusfremontii*, *Rhusovata*, *Baccharissergiloides*, *Quercus* sp., *M. G. Simpson 2794 with K. Hasenstab*, *M. Silveira*, *and L. Simpson*, 7 April 2007 (**SDSU**17573!); • Pacific Coast Trail, ca. 0.1 mile north of parking area at Hwy 74., California, Riverside Co., 33.56416, -116.57673, 1499 m elevation, beneath Adenostomafasciculatum. Adjacent to open *Adenostomasparsifolium* - *Adenostomafasciculatum* - *Ceanothusgreggii* scrub, coarse sand, associates: *Adenostomasparsifolium*, *Adenostomafasciculatum*, *Ceanothusgreggii*, *M. G. Simpson 3080 with Lori Simpson*, 26 April 2009 (**SDSU**21885!); • Pacific Coast Trail, ca. 0.6 mile north of parking area at Hwy 74., California, Riverside Co., 33.56977, -116.57572, 1526 m elevation, side of trail. Open *Adenostomasparsifolium* - *A.fasciculatum* - *Ceanothusgreggii* scrub., coarse sand, associates: *Adenostomasparsifolium*, *Adenostomafasciculatum*, *Ceanothusgreggii*, *M. G. Simpson 3085 with Lori Simpson*, 26 April 2009 (**SDSU**21647!); • Anza Borrego Desert State Park. Wash of Bitter Creek Canyon, ca. 1.0 mile north of Grapevine Mountain peak., California, San Diego Co., 33.13444, -116.46798, 739 m elevation, along base of canyon wall rocks of wash, open scrub of desert wash, gravelly sand, associates: *Agavedeserti*, *Chenopodium* sp., *Enceliafarinosa*, *Opuntiabasilaris*, *Senegaliagreggii*, *M. G. Simpson 3790 with Amanda Everett*, *Andy Siekkinen*, *Lee Ripma*, *Matt Newcomb*, 15 February 2014 (**SDSU**20519!); • Anza Borrego Desert State Park, Cool Canyon, ca. 1,000 m west-southwest of parking area at end of dirt road, ca. 1.8 km east-southeast of Granite Mountain peak., California, San Diego Co., 33.04362, -116.44427, 869 m elevation, desert succulent scrub, brown, sandy loam, associates: *Juniperuscalifornica*, *Cylindropuntiaganderi*, *Prunusfremontii*, *Enceliafarinosa*, *Bahiopsisparishii*, *M. G. Simpson 3795 with SDSU Taxonomy of California Plants Class*, 29 March 2014 (**SDSU**23021!); • Anza Borrego Desert State Park, Bitter Creek Canyon, ca. 2.3 kilometers southwest of Grapevine Canyon Road (dirt)., California, San Diego Co., 33.13402, -116.47022, 745 m elevation, wash of canyon, desert succulent scrub, silty, coarse sand, associates: *Prosopisglandulosa*, *Enceliafarinosa*, *Chenopodiummurale*, *Bromusrubens*, *M. G. Simpson 3800 with Makenzie E. Mabry*, 3 April 2014 (**SDSU**23020!); • Anza Borrego Desert State Park, Culp Valley, along hiking trail at camping area., California, San Diego Co., 33.22376, -116.45312, 1033 m elevation, flat, coarse sand/fine gravel, organic material beneath, associates: *Rhusovata*, *Ziziphusparryivar.parryi*, *Cylindropuntiaganderi* scrub, *M. G. Simpson 3856 with Makenzie Mabry & Andy Siekkinen*, 1 April 2015 (**SDSU**23271!); • Culp Valley, ca. 0.1 mile along dirt road, to parking lot at left, then 10 meters west of northern end of lot., California, San Diego Co., 33.22209, -116.45791, 1014 m elevation, desert transtion vegetation, brownish, gravelly sand, growing adjacent to boulder, associates: *Prunusfremontii*, Ziziphusparryivar.parryi, *M. G. Simpson 4186 with Jepson 2019 workshop members*, 14 April 2019 (**SDSU**22965!); • San Gabriel Mountains, east side of Cabin Canyon, c. 200 m north of canyon mouth, west of Aliso Cyn. Rd., 0.5 mi west of Angeles Forest Hwy, California, Los Angeles Co., 34.4169444, -118.0925, 1183 m elevation, shallow canyon bottom with flowing stream; chaparral dominant, associates: *Ceanothus leu*., *Adenostoma f*., *Quercus wis*., *Prunus ili*., *Eriodictyon tri*., with *Layia gla*., *Phacelia fre*., *Calyptridium mon*., *Penstemon cen*., *Malacothrix gla*., *Camissoniopsis hir*., *Bromus tec*., *Antirrhinum cou*., *Cirsium occ*., *Chaenactis gla*., etc, *R. G. Swinney 14663*, 12 May 2011 (**RSA**0091168!, **SDSU**22957!); • San Gabriel Mountains, 0.3–0.5 miles south of Aliso Canyon Road, 2 miles west of Angeles Forest Hwy., nameless cyn. To the east of Beartrap Cyn., California, Los Angeles Co., 34.42778, -118.11639, 1066 m elevation, Station Fire burn of 2009, shallow ravine and adj. slopes, chaparral, associates: *Adenostoma f*., *Prunus i*., *Lonicera su*., *Rhus ar*., *Cercocarpus b*., also w/ *Opuntia ba*., *Malacothamnus fr*., *Lupinus bi*., *Camissonia s.*, *Uropappus*, *Mentzelia co.*, *Delphinium pat.*, *Phacelia fr.*, *Leptosiphon a*., etc, *R. G. Swinney 14845*, 26 May 2011 (**RSA**0092147!, **SDSU**22955!); • San Gabriel Mountains: Colby Ranch (Camp Colby), S end of Inspiration Canyon tr., in a N-flowing tributary of upper Coldwater Cyn., California, Los Angeles Co., 34.29861, -118.11361, 1226 m elevation, Station Fire burn of ‘09, canyon wih slopes; chaparral, coarse granite and fine brown loam, associates: *Ceanothus*, *Eriodictyon*, *Quercus*, *Pinus*, *Nicotiana*, *Eschscholzia*, *Phacelia*, *Adenostoma*, *Mimulus*, *Chaenactis*, *Keckiella*, *Calyptridium*, *Salvia*, *Galium*, *Penstemon*, *Argemone*, *Erhendorferia*, *Dendromecon*, *Eriogonum*, *Mentzelia*, *Solanum*, *R. G. Swinney 15440*, 7 July 2011 (**RSA**0091783!, **SDSU**22954!, **UCR**0004166!); • San Gabriel Mountains: Colby Ranch (Camp Colby), S end of Inspiration Canyon tr., in a N-flowing tributary of upper Coldwater Cyn., California, Los Angeles Co., 34.29861, -118.11361, 1226 m elevation, Station Fire burn of ‘09, canyon wih slopes; chaparral, coarse granite and fine brown loam, associates: *Ceanothus*, *Eriodictyon*, *Quercus*, *Pinus*, *Nicotiana*, *Eschscholzia*, *Phacelia*, *Adenostoma*, *Mimulus*, *Chaenactis*, *Keckiella*, *Calyptridium*, *Salvia*, *Galium*, *Penstemon*, *Argemone*, *Erhendorferia*, *Dendromecon*, *Eriogonum*, *Mentzelia*, *Solanum*, *R. G. Swinney 15442*, 7 July 2011 (**RSA**0091784, **SDSU**22946!, **UCR**0039547); • San Gabriel Mountains: Strawberry Peak summit (Chilao Flat)., California, Los Angeles Co., 34.28389, -118.12028, 1869 m elevation, Station Fire burn of 2009, entire summit burned; resprouting chaparral, associates: *Eriodictyontrichocalyx*, *E.parryi*, *Quercuswislizeni*, *Q.chrysolepis*, *Penstemoncentranthifolius*, *Eriastrumdensifolium*, *Ericamerianausiosa*, *Camissoniopsishirtella*, *Bromustectorum*, *B.carinatus*, *Eriophyllumconfertiflorum*, *Cryptanthamuricata*, *Garryaflavescens*, *Tauschiaparishii*, *Eriogonumsaxatile*, *Orobanchefasciculata*, *Sidothecatrilobata*, etc, *R. G. Swinney 15527*, 21 July 2011 (**RSA**0089514, **SDSU**22959!, **UCR**0004165); • San Gabriel Mts., n. e. boundary ridge top of San Dimas Experimental Forest, c. 0.14 miles s.w. of summit at far s.e. end of ridge, 1.7 air miles w. of Cow Cyn. Saddle at Glendora Ridge Rd. to 1.8 land miles ESE along SDEF boundary ridge to ridge top at 5595 ft. (1706 m.), and east via level ridge connection to ridge top at 5560 ft., 04 air miles NNE of Sunset Pk. summit. This is the highest elev. within SDEF, Mt. Baldy Quad, California, Los Angeles Co., 34.209722, -117.695278, 1695 m elevation, Ridge top, chaparral, Big Cone Spruce/*Quercus* woodland, associates: *Pseudotsugamacrocarpa*, *Quercuswislizeni subsp. frutescens*, *Q.chrysolepis*, *Ceanothuscrassifolius*, Eriogonumfasciculatumsubsp.foliolosum, *Bromustectorum*, *R. G. Swinney 18319*, 4 May 2016 (**SDSU**23973!); • North of Azusa, WCA’s Azusa Foothills Open Space, ridgetop overlooking mouth of San Gabriel Cyn., within 50 meters of Vasquez Ranch Flagpole, last burned 2014 - Cobly Fire, 0.55 air miles n. of jct. of Sierra Madre Ave. and Hilltop Dr., Azusa Quad 7.5’, California, Los Angeles Co., 34.156355, -117.904528, 450 m elevation, ridge top, coastal sage scrub and chaparral surrounding previously slightly-disturbed area, granitic-loam, associates: *Malosmalaurina*, *Bromusdiandrus*, *Enceliafarinosa*, *Artemisiacalifornica*, *Hirschfeldiaincana*, Malacothrixsaxatilisvar.tenuifolia, *R. G. Swinney 24723A*, 5 May 2021 (**SDSU**25595!, **UCR**0164808); • San Bernardino Mountains: unnamed ridgetop (7,582’) and adjacent slope just W of Heart Bar Peak and SE of Cienaga Seca Creek., California, San Bernardino Co., 34.1667, -116.775, 2222 m elevation, ridgetop recently bulldozed for firebreak; adjacent N and W slopes w/ *Quercuschrysolepis* and *Pinusjeffreyi*, etc., *Quercuschrysolepis* and *Pinusjeffreyi*, etc., *S. D. White 13324 with Marissa Caringella*, 24 July 2009 (**RSA**752962!, **UC**1949977, **UCR**0004334, **UCSB**029502); • Peninsular Ranges: Santa Rosa Mountains, California, Riverside Co., 33.539*, -116.457*, 2310 m elevation, *R. S. Woglum 3072*, 12 June 1941 (**RSA**611564!); • San Bernardino Mountains: Unnamed canyon and ridgeline immediately SW of Cienega Seca (Blue Sky Meadow), California, San Bernardino Co., 34.1833, -116.725, 2476 m elevation, yellow pine forest with somes areas of pinyon/juniper woodland, *J. M. Wood 948 with Dustin Ray*, 18 June 2009 (**RSA**752329!); • San Bernardino Mtns., Santa Ana River, unnamed canyon and ridgeline E of Wildhorse Canyon, N of Hwy 38, California, San Bernardino Co., 34.17083, -116.8, 2100 m elevation, dry canyon wash, riparian woodland and adjacent uplands, *J. M. Wood 1013 with Dustin Ray*, 30 June 2009 (**RSA**750733!); • Borrego Palm Canyon, California, San Diego Co., 33.276836*, -116.426169*, 322 m elevation, *F. Youngberg 103*, 11 April 1935 (**POM**209514!).

## ﻿Discussion

**Figure 4.**
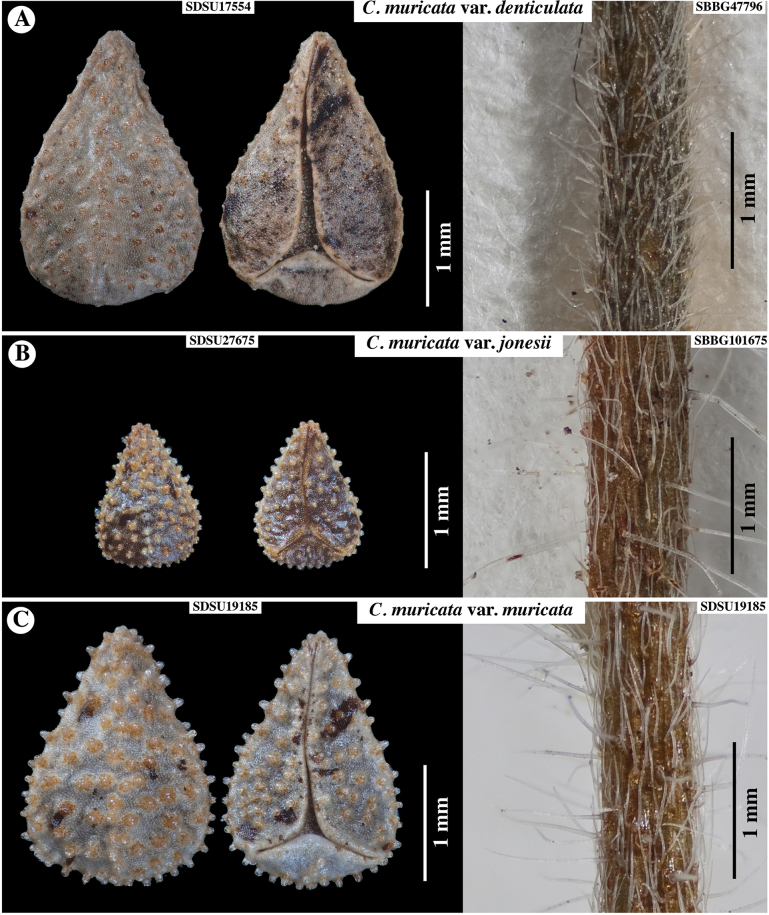
Nutlets (dorsal view at left, ventral at right) and stem morphology of the three recognized varieties of *C.muricata*, series *Muricatae***A**Cryptanthamuricatavar.denticulata**B**Cryptanthamuricatavar.jonesii**C**Cryptanthamuricatavar.muricata. Accession numbers of specimens sampled indicated. Note variation in nutlet sculpturing and stem vestiture that is strigose and hispid.

*Cryptanthaacrimuricata* is morphologically distinctive, but is an obvious close relative of *C.muricata*, given its similarity in fruiting calyx shape (ovoid), style length (typically longer than the nutlets), nutlet number (four per fruit), nutlet shape (ovate, typically with a dorsal ridge, this sometimes obscure), and nutlet sculpturing pattern (tuberculate to muricate). As noted by Johnston (1925), *C.muricata* has the edges of the nutlets thickened to form a “beaded” or “toothed” margin (Fig. 4), this feature also seen in *C.acrimuricata* (Fig. 2).

We confirmed that the type specimens of the three heterotypic varietal synonyms of *C.muricata* (Table 1) did not conform to the morphology of *C.acrimuricata* and thus feel confident that our new species had not been previously named. We considered treating *C.acrimuricata* as a fourth variety of *C.muricata*. However, we felt justified in treating it as a separate species for two reasons. First, it is different from the other species in two major features: stem vestiture and nutlet sculpturing, characters which, based on our observations, appear to be stable. Second, we have no evidence that it is more closely related to one or more of the varieties of *C.muricata* than to other species of the complex (see below). In fact, future studies may support elevation of one or more varieties of *C.muricata* to species status (see below). Thus, we think it more stable to recognize this unique taxon as its own species, pending more detailed molecular phylogenetic studies. *Cryptanthamuricata*, with its three currently recognized varieties, is a taxonomically challenging complex. Preliminary data indicate that one or more of the currently recognized varieties—C.m.var.denticulata (Greene) I.M.Johnst. (Fig. 4A), C.m.var.jonesii (A.Gray) I.M.Johnst. (Fig. 4B), and C.m.var.muricata (Fig. 4C)—may eventually be elevated to species status or split into more than one taxon. The complex is currently under study by members of the Amsinckiinae Working Group (2024).

The distributions of *Cryptanthaacrimuricata* and of the three varieties of *C.muricata* show some discrete geographic clustering but also overlap in some regions. *Cryptanthaacrimuricata*, as cited before, occurs in the eastern part (escarpment) of the Peninsular Ranges of California and Baja California, Mexico, in higher elevations of the Mohave and Sonoran Deserts of California and Arizona, and in localities of the eastern and central Transverse Ranges and southern Sierra Nevada of California (Fig. 3). Cryptanthamuricatavar.jonesii is most common in the western part of the Peninsular Ranges westward to the coast, as well as in parts of the Transverse Ranges. Cryptanthamuricatavar.muricata is most common in the western part of the Transverse Ranges, Central Coast Ranges, and northward to the San Francisco Bay area of California. Cryptanthamuricatavar.denticulata is mostly restricted to the southern and central Sierra Nevada but also has populations in the western Transverse Ranges (Fig. 3).

**Figure 5.**
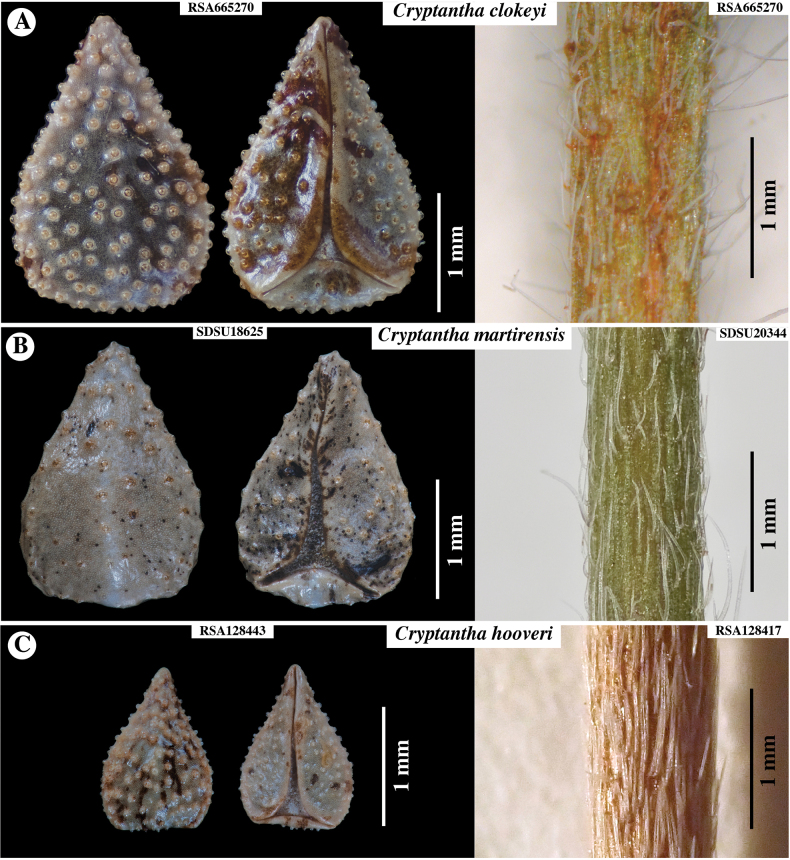
**A, B** Nutlets (dorsal view at left, ventral at right) and stem morphology of three additional *Cryptantha* species of series *Muricatae***A***Cryptanthaclokeyi***B***Cryptanthamartirensis***C***Cryptanthahooveri*, tentatively placed in series. Accession numbers of specimens sampled indicated.

We believe *Cryptanthaacrimuricata* should be classified in series *Muricatae* of Johnston (1925). This subgeneric group was diagnosed by him as “Nutlets 4, verrucose or coarsely tuberculate, triangular-ovate, decidedly homomorphous, back obtuse and bearing a suggestion of a medial ridge, with sides evidently angles and beaded; style usually surpassing the nutlets though rarely only equally them” (Johnston 1925: 69–70). Another possible feature that could be added as a diagnostic feature of this series is a style extending beyond the nutlets in fruit. Series *Muricatae* originally included only *C.muricata*, with its currently recognized three varieties. Later, Johnston (1939) suggested that *C.clokeyi* I.M.Johnst. (Fig. 5A) be placed in *Muricatae*. Subsequently, Simpson and Rebman (2013: 41) stated that their newly described *C.martirensis* M.G.Simpson & Rebman (Fig. 5B) “...may be tentatively placed in sectionMuricatae; however, molecular phylogenetic studies are needed to verify the monophyly of this group.” Indeed, the subsequent molecular phylogenetic analyses of Simpson et al. 2017b and Mabry and Simpson 2018 confirmed the composition of three members of series *Muricatae*, as it is delimited here (Table 1). In both studies C.muricatavar.muricata is sister to *C.clokeyi*, with these two sister to *C.martirensis*, all with strong support.

One species of *Cryptantha* whose relationships has been a bit of a mystery is *C.hooveri* I.M.Johnst. In the protologue for *Cryptanthahooveri*, Johnston (1937: 24) stated “I can suggest no close relative for this very distinct species.” *Cryptanthahooveri* does have a number of unique features, such as clustered, head-like cymes and bracteate flowers, the bracts very narrow (“thread-like”). Subsequently, however, Johnston (1939: 388) suggested that *Cryptanthaclokeyi* is “perhaps most closely related to *Cryptanthahooveri* Johnst. of the Sierran foothills of central California.” *Cryptanthahooveri* does resemble *C.clokeyi* in having ovate to deltate nutlets with a similar, tuberculate sculpturing (Fig. 5C), and it does have the elongate style, extending beyond the nutlets in fruit, characteristic of series *Muricatae*. However, aside from its unique features, *C.hooveri* differs from *C.clokeyi* in having calyces and nutlets about half the size of the latter species (Fig. 5B, C). Unfortunately, *C.hooveri* is presumed extinct (CNPS Inventory 2024), having been last collected in 1939 and not observed since.

These three additional members of series *Muricatae*—*C.clokeyi*, *C.hooveri*, and *C.martirensis*—are somewhat restricted in range (not mapped here), occurring, respectively, in regions of the Mohave Desert and Transverse Ranges, in the Central Valley, and in the San Pedro Martir mountains of Baja California, Mexico. They are not sympatric with *C.acrimuricata* or varieties of *C.muricata*.

The subgeneric classification of *Cryptantha* is an ongoing project of research (Amsinckiinae Working Group 2024). Although most of Johnston’s series do not correspond to monophyletic groups (Mabry and Simpson 2018), series *Muricatae*, as expanded here, does and should be accepted (Table 1). Interestingly, from the aforementioned molecular phylogenetic studies of Simpson et al. 2017b and Mabry and Simpson 2018, series *Muricatae* itself appears to be sister to a modified series *Maritimae* (Johnston 1925), named after *Cryptanthamaritima*. The two series, *Muricatae* and *Maritimae*, were together termed the “*Maritimae* clade” in those studies. Further studies are needed to refine these relationships. It seems clear, however, that based on the phylogenetic studies to date, our new species should be placed in series *Muricatae* of the *Maritimae* clade, the latter a monophyletic group separate from the bulk of the genus *Cryptantha*, which may in the future warrant a formal name (Simpson et al. 2017b, Mabry and Simpson 2018).

Given the evidence for the relationships of *Cryptanthaacrimuricata*, we here present a key to our accepted members of *Cryptantha* series *Muricatae* (tentatively including *C.hooveri*).

### ﻿Key differentiating *Cryptantha* taxa within series *Muricatae* (See Figs 1, 2, 4, 5 for images of nutlets and other diagnostic features)

**Table:** 

1	Cymules tightly clustered, head-like, only lowest flowers in each cymule developing fruit; flower bracts mostly present, filiform in shape	***C.hooveri***
–	Cymules elongate at maturity, most flowers developing fruit; flower bracts absent	**2**
2	Calyx 5–9(–10) mm long; nutlets without dorsal ridge, (1.8–)2.4–2.8(–3) mm long	***C.clokeyi***
–	Calyx 2–5 mm long; nutlets with dorsal ridge, 1.1–2.2 mm long	**3**
3	Corolla limb showy, 3–8 mm wide	**C.muricatavar.muricata**
–	Corolla limb generally inconspicuous, 1–2(3.5) mm wide	**4**
4	Nutlet tubercles conic in shape, very densely spaced, often whitish, intervening surfaces often shiny, appearing shellacked; stems mostly strigose, occasionally also spreading hispid, appearing whitish to gray-green in color	***C.acrimuricata***
–	Nutlet tubercles terete to rounded in shape, less densely spaced, brownish, intervening surfaces generally not shiny; stems strigose and densely hispid, generally green or yellow-green in color	**5**
5	Nutlets 1.1–1.3(1.9) mm long, muricate, tubercles generally elongate	**C.muricatavar.jonesii**
–	Nutlets 1.8–2.2 mm long, tuberculate, tubercles generally low, rounded	**6**
6	Primary stem axis 11–53 cm long, not obviously different from secondary axes; inflorescence cymules, including stalk, 12–140 mm long	**C.muricatavar.denticulata**
–	Primary stem axis 35–68 cm long, prominent, elongate, virgate; inflorescence cymules, including stalk, 5–10 mm long	***C.martirensis***

## ﻿Conclusions

Research on the taxonomy and phylogenetic relationships of *Cryptantha* and other members of the “Popcorn Flowers” (subtribe Amsinckiinae) is an ongoing project of the Amsinckiinae Working Group (2024). We point out that new species of *Cryptantha* continue to be discovered; in addition to this new taxon, five new species and one new variety have been described in the last 11 years. *Cryptanthaacrimuricata* was originally recognized as a distinct form from the study of herbarium specimens and subsequently from field collections. The majority of herbarium records of this new species had been housed in herbarium cabinets for years, most identified as *C.muricata*. As we have emphasized before, the discovery of this new species highlights the need for continued field collections of plant specimens, their storage and databasing in herbaria, and the study of those specimens by scientific experts. There are undoubtedly many more species yet to be named, some lying in herbarium collections, waiting to be discovered (Bowdler 2010).

## Supplementary Material

XML Treatment for Cryptantha acrimuricata

## ﻿Additional information

### Author ORCIDs

Michael G. Simpson https://orcid.org/0000-0002-6197-2132

James M. André https://orcid.org/0009-0000-0433-7493

### Data availability

All of the data that support the findings of this study are available in the main text.
